# Influence of Material Properties on TiO_2_ Nanoparticle Agglomeration

**DOI:** 10.1371/journal.pone.0081239

**Published:** 2013-11-25

**Authors:** Dongxu Zhou, Zhaoxia Ji, Xingmao Jiang, Darren R. Dunphy, Jeffrey Brinker, Arturo A. Keller

**Affiliations:** 1 Bren School of Environmental Science and Management, University of California Santa Barbara, Santa Barbara, California, United States of America; 2 University of California Center of Environmental Implications of Nanotechnology, University of California Los Angeles, Los Angeles, California, United States of America; 3 Center for Micro-Engineered Materials and Department of Chemical Engineering, University of New Mexico, Albuquerque, New Mexico, United States of America; 4 Sandia National Laboratory, Albuquerque, New Mexico, United States of America; University of California, Merced, United States of America

## Abstract

Emerging nanomaterials are being manufactured with varying particle sizes, morphologies, and crystal structures in the pursuit of achieving outstanding functional properties. These variations in these key material properties of nanoparticles may affect their environmental fate and transport. To date, few studies have investigated this important aspect of nanoparticles' environmental behavior. In this study, the aggregation kinetics of ten different TiO_2_ nanoparticles (5 anatase and 5 rutile each with varying size) was systematically evaluated. Our results show that, as particle size increases, the surface charge of both anatase and rutile TiO_2_ nanoparticles shifts toward a more negative value, and, accordingly, the point of zero charge shifts toward a lower value. The colloidal stability of anatase sphere samples agreed well with DLVO theoretical predictions, where an increase in particle size led to a higher energy barrier and therefore greater critical coagulation concentration. In contrast, the critical coagulation concentration of rutile rod samples correlated positively with the specific surface area, i.e., samples with higher specific surface area exhibited higher stability. Finally, due to the large innate negative surface charge of all the TiO_2_ samples at the pH value (pH = 8) tested, the addition of natural organic matter was observed to have minimal effect on TiO_2_ aggregation kinetics, except for the smallest rutile rods that showed decreased stability in the presence of natural organic matter.

## Introduction

Given the accelerating production of existing and emerging engineered nanoparticles (ENPs), the accidental spill and use-phase or end-of-product-life release of nanoparticles into the environment may be inevitable [1]–[3]. In fact, a few studies have already reported detectable levels of TiO2 nanoparticles in a wastewater treatment plant and in natural water streams [4]–[6]. To accurately assess the environmental distribution, the major sinks, and the ecological risks of ENPs, a comprehensive understanding of how ENPs behave in the aqueous environment is imperative [1], [2], [7]–[9].

The fate and transport of ENPs in the aqueous environment is controlled by both the chemistry of the aqueous systems and the material properties of the ENP [2], [10]–[12]. In recent years, the effect of solution chemistry on the aggregation of mostly spherical ENPs has been extensively studied [13]–[19] and is relatively well understood. For instance, pH alters the colloidal stability of the ENPs system by modulating the protonation/deprotonation equilibrium and further altering the electrostatic repulsion [13], [17]. Indifferent electrolytes compress the nanoparticle electric double layer and reduce the energy barrier [20], [21]. The presence of natural organic matter, depending on the concentration, can either stabilize nanoparticles by providing additional electrostatic repulsion and/or steric hindrance, or bridge multiple particles and enhance aggregation [16], [19]. Our recent study revealed that natural clay minerals can coagulate either positively or negatively charged nanoparticles due to their edge-face charge heterogeneity [22].

On the other hand, only until recently limited studies started to investigate the effect of intrinsic material properties of ENPs, such as particle size, morphology, crystal structure, and dopants on ENPs' aggregation and mobility [2], [23]. Kobayashi et al. demonstrated that an additional repulsive force appears on silica surfaces as particle size decreases [24]. He et al. showed that larger hematite nanoparticles (65 nm and 32 nm) were more stable than smaller ones (12 nm), which was qualitatively explained by DLVO theory [25]. Mulvihill et al. investigated the effects of three stabilizing agents on the colloidal stability of CdSe nanoparticles (4, 6, and 8 nm spheres and 2.9×24 nm rods) [26], and they found capping ligand dissociation to be the primary nanoparticle aggregation mechanism. In addition, the critical coagulation concentrations of four different CdSe nanoparticles were found to be linearly correlated to their specific surface area [26]. Liu et al. reported that 50 nm anatase spheres and 10×40 nm rutile rods settled more slowly than 5 and 10 nm anatase nanospheres. Differences in the amounts of sulfur and phosphate impurities introduced during the synthesis process determine the stability of TiO_2_ spheres and rods [27]. These studies suggest that material properties such as particle size, capping ligand, and impurities are important parameters affecting nanoparticles' aqueous stability. However, a systematic study on the role of intrinsic properties of nanoparticle aggregation where particle size is progressively varied and crystal structure is controlled is lacking.

The goal of this study was to investigate the influence of particle size, morphology, and crystal structure in TiO_2_ nanoparticle aggregation. The two most abundant polymorphs of TiO_2_ are rutile and anatase, both crystalize in the tetragonal system. Rutile is the stable phase, and anatase is metastable [28]. Rutile and anatase of increasing size were synthesized and their colloidal stability was characterized by means of electrophoretic mobility and light scattering. We present results on particle charge, critical coagulation concentrations, and absolute doublet formation rates. We found that no single material property was a determining factor that controls TiO_2_ aggregation; rather, a combination of various material parameters needs to be considered to predict nanoparticle aggregation.

## Materials and Methods

### 2.1 Materials

Ten TiO_2_ samples, five rutile rods (designated RR) and five anatase spheres (designated AS), with varying sizes were synthesized via a hydrothermal approach. In a typical synthesis of spherical anatase NPs (AS samples), 1.34 g of amorphous titanium dioxide (NanoActive, Nanoscale Corp.) was added to 43 g of 1 M H_2_SO_4_ and heated to 230°C for 24 hours in a Parr bomb. For rutile NPs rods (RR samples), 23.7 g of TiCl_4_ was dissolved in 50 ml of 37% HCl; 6.0 g of this solution was added to 11.7 g of 1 M HCl and then heated to 200°C for 24 hours, again in a Parr bomb. NP size for both rutile and anatase was varied by control of the precursor/acid ratio, with increased precursor concentration yielding progressively larger particle sizes. To remove residual salt, sample suspensions were dialyzed (MWCO 12–14k, Spectrum Laboratories, CA) against de-ionized water until the conductivity inside and outside of the membrane were identical.

Sample crystal structure was characterized by X-ray Diffraction (XRD) (X'pert Powder, PANalytical, the Netherlands). Particle morphology and size were assessed by transmission electron microscopy at 80 kV (JEOL 1230, JEOL, Japan). TEM samples were prepared by placing a drop of the TiO_2_ suspension on a 200 mesh copper grid (Ted Pella, CA) and allowing it to air dry overnight. ImageJ software (NIH, USA) was used to determine the particle dimensions. The number-weighted dimensions (diameter for spheroids; length × width for rods) are determined by measuring 100 randomly selected nanoparticles on the TEM images, and the surface areas are calculated assuming a sphere shape for anatase samples and a cylinder shape (length as the cylinder height and width as the base diameter) for rutile samples (Table 1). For comparison purposes, all RR samples had an aspect ratio of around 3.5–4.5. The RR samples were relatively monodisperse, with a coefficient of variation (CV) in the range of 0.2–0.35, except RR4 which has a much higher polydispersity (CV≈0.6). The number-weighted diameter of AS particles ranged between 6–150 nm. The CV values were 0.2–0.3.

**Table 1:** Measured properties of the TiO_2_ samples.

Sample	Primary Particle Dimension (nm)^a^	Specific surface area (m^2^/g) b	Crystal Struture	CCC (mM), without NOM	CCC (mM), with 10 mg/L NOM
RR1	15±5×4±1	236.4	rutile	240	80
RR2	41±14×10±2	78.8	rutile	77	65
RR3	121±33×29±7	31.5	rutile	65	- c
RR4	201±122×40±32	23.6	rutile	75	40
RR5	193±64×52±12	18.2	rutile	25	30
AS1	6±2	59.1	anatase	25	40
AS2	11±3	32.2	mixture of rutile and anatase	25	15
AS3	38±7	9.3	anatase	30	50
AS4	54±17	6.6	anatase	65	50
AS5	152±43	2.3	anatase	100	25

a primary particle dimensions are determined by measuring 100 randomly-chosen particles on TEM images.

b specific surface area is calculated based on the particle dimensions, assuming rutile rods as cylinders and anatase spheres as perfect spheres. The density of TiO2 was 4.23×10^6^ g/m^3^.

c CCC of RR3 with NOM appeared to be below the lowest tested electrolyte concentration, thus no CCC is reported here.

Suwannee River natural organic matter (NOM) was purchased from the International Humic Substance Society (IHSS, GA, USA). A 200 mg/L stock was prepared by dissolving NOM in deionized water. All reagents used in this study were analytical grade. NaCl (Sigma-Aldrich) was used as the indifferent electrolyte. Borate buffer was used to maintain a constant pH = 8.0. HCl (0.1 M and 0.01 M, EMD Chemicals Inc.) and NaOH (0.1 M, Fisher Scientific) were used as titrants for point of zero charge titration. All the solutions were filtered via 0.22 mm PVDF filters to avoid potential interference in the light scattering experiments.

### 2.2 Electrophoretic mobility measurements

Electrophoretic mobility was measured on a Malvern Zetasizer coupled with a MPT-2 autotitrator (Malvern, UK). 0.1 M, 0.01 M HCl and 0.1 M NaOH were used as titrates. A 120 s equilibribration time was allowed before each measurement. At each pH value, data were collected in triplicate.

### 2.3 Aggregation kinetics

The detailed procedure for measuring aggregation kinetics is described elsewhere [22]. Briefly, predetermined amounts of buffer stock (20 mM), NaCl stock (1 M, 0.1 M, and 0.01 M), NOM (1 g/L, when the effect of NOM was investigated), and deionized water were mixed together to make up 0.9 mL total volume at the desired pH and ionic strength. This mixture was added into 0.1 mL of a specific TiO_2_ stock suspension immediately before the aggregation kinetics measurements. The hydrodynamic diameter of the suspension was monitored by dynamic light scattering (Malvern Zetasizer Nano, UK) as a function of time. The detection angle was 90°, and the laser wavelength was 633 nm. The cumulant algorithm was used to calculate the hydrodynamic diameter. The measurements lasted until either the hydrodynamic diameter of the sample doubled or the measurement duration reached 1 hr, whichever criterion was met first.

The slope of the early-stage aggregation (arbitrarily fixed as the time from a_0_ to 1.5×*a_0_*) curve was then used to determine the doublet formation rate according to [14]:
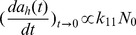
here *a_h_(t)* is the hydrodynamic diameter of agglomerates as a function of time *t*, *N_0_* is the initial number concentration of primary particles, and *k_11_* is the doublet formation rate. The attachment efficiency (α) - salt concentration plot is typically characterized by two regimes, the reaction- and diffusion-limited cluster aggregation regimes (RLCA and DLCA). α increases with salt concentration in the RLCA regime; while it is independent to salt concentration in the DLCA regime. The turning point between the two regimes is called the critical coagulation concentration (*CCC*).

## Results and Discussion

### 3.1. Nanoparticle characterization

**Figure 1:**
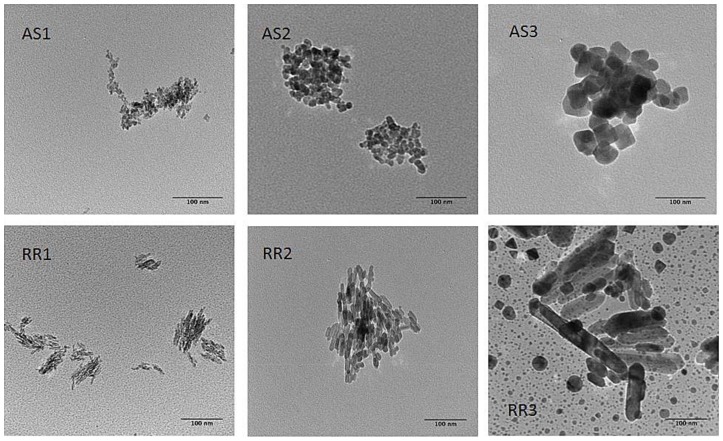
Representative TEM images of rutile rods and anatase spheroids.

**Figure 2:**
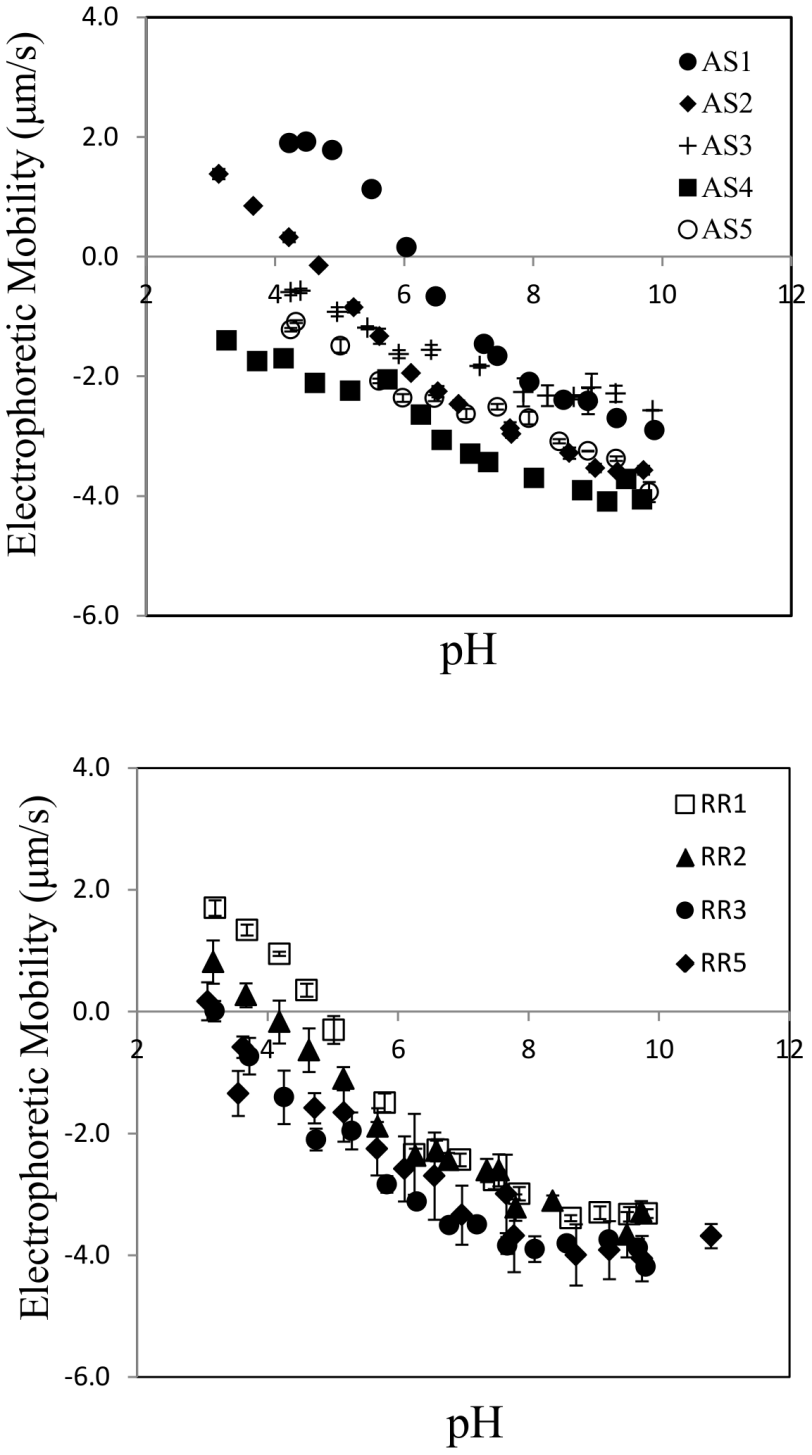
Electrophoretic moblity of anatase spheres (a) and rutile rods (b) as a function of pH. A general trend of PZC shift toward a lower pH can be observed for both anatase spheres and rutile rods.

Morphology and surface charge characteristics of the TiO_2_ samples were quantified by TEM (Figure 1 and File S1) and electrophoresis, respectively. Regardless of the morphology, crystal structure, and size, all TiO_2_ samples showed typical amphoteric charging patterns [29]. Three types of groups (singly coordinated Ti_3_O^0^, doubly coordinated Ti_2_O^2/3−^, and triply coordinated TiO^4/3−^) with varying H^+^/OH^−^ affinity constants (pK) exist on the TiO_2_ surface [30]. Due to the extremely low pK value (−7.5) for TiO^4/3−^, the singly and doubly coordinated groups determine the actual TiO_2_ surface charge. The point of zero charge (PZC) values ranged from pH 3 to 6. These results are in good agreement with previously published values [13], [27], [30]–[32]. A correlation between the electrophoretic mobility and particle size was observed for both anatase spheres and rutile rods (Figure 2). The point of zero charge (PZC) shifts toward a lower pH value with increasing particle size for the same aspect ratio. This trend was observed experimentally for TiO_2_ anatase spheres by others [27], [33]. Using a corrected Debye-Huckel theory and Monte Carlo simulation, Abbas et al. showed theoretically that such a size-dependence of surface charge exists for metal oxide nanoparticles [34]. It is suggested that, as particle size decreases, the curvature of nanoparticles approaches the same length scale as the hydrated ions, which enables the counterions to screen the surface sites from all directions rather than only one-half of the space in the case of a planar wall.

### 3.2. Aggregation kinetics

**Figure 3:**
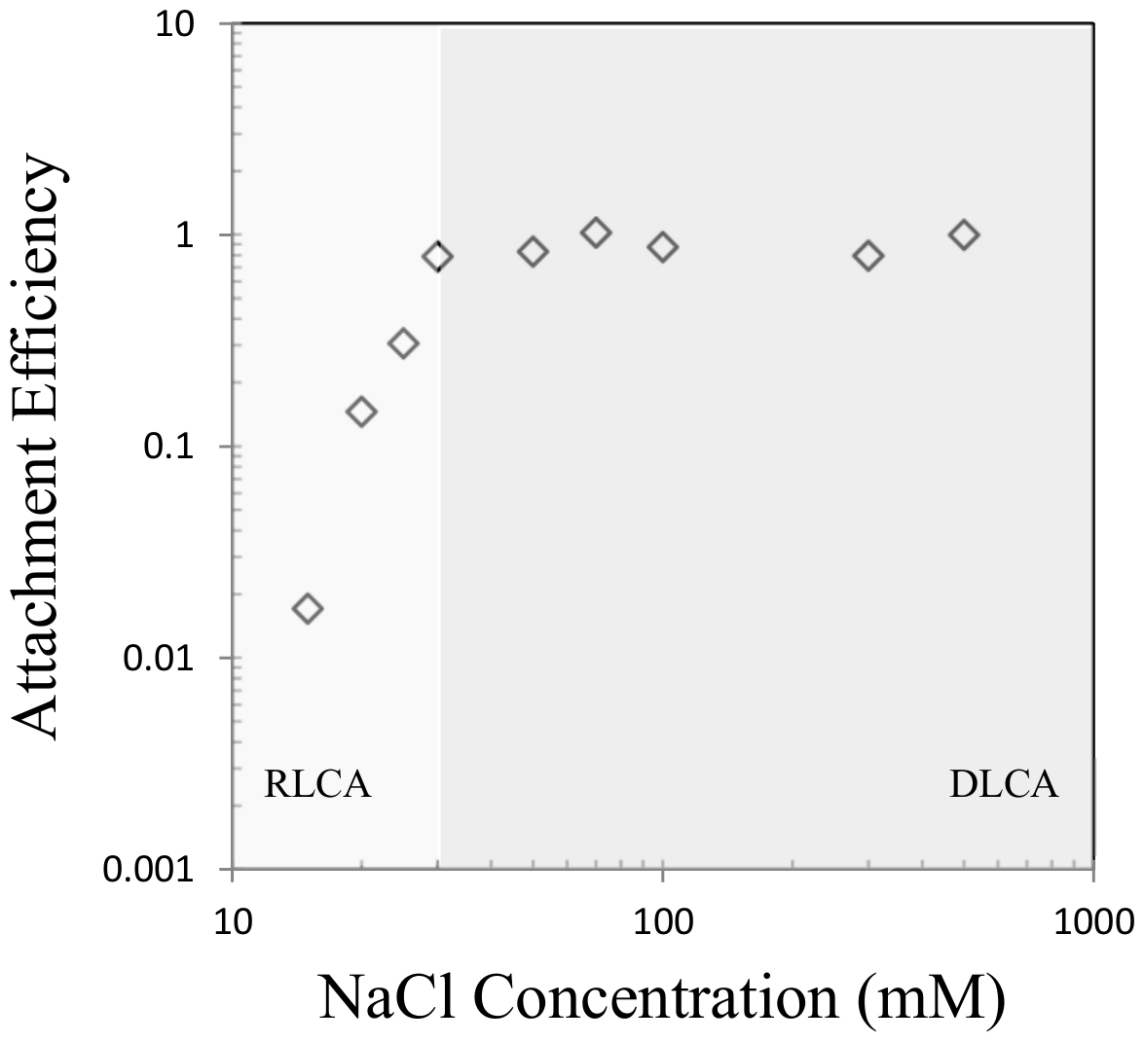
The attachment efficiency as a function of NaCl concentration for AS3 (anatase sphere). A reaction-limited cluster aggregation regime (RLCA, left region) and a diffusion-limited cluster aggregation regime (DLCA, right region) can be observed.

The aggregation kinetics of the various TiO_2_ nanoparticles was examined over a wide range of NaCl concentrations (1–500 mM). A representative aggregation kinetics curve is shown in Figure S2 in File S1 and an attachment efficiency - electrolyte concentration plot is shown in Figure 3 (to avoid redundancy, additional aggregation kinetics curves are not shown). The reaction limited cluster aggregation (RLCA) and diffusion limited cluster aggregation (DLCA) regimes can be identified in the stability plots of every TiO_2_ sample. It appears that the electrostatic and van der Waals interactions control the TiO_2_ aggregation process even for diverse morphologies [13], [18], [27], [29]. At low NaCl concentrations, electrostatic repulsion dominates the inter-particle interaction and aggregation occurs at a relatively slow rate. As electrolyte concentration increases, the electrostatic repulsion is suppressed due to the electric double layer (EDL) compression, and the aggregation is accelerated. Once the electrolyte concentration is high enough to completely eliminate the energy barrier, van der Waals attraction starts to dominate and most collisions between TiO_2_ nanoparticles lead to attachment. This electrolyte concentration, called critical coagulation concentration (CCC), can serve as an index to compare nanoparticle suspension stability. The CCC values are summarized in Table 1.

### 3.3. Influence of material properties

**Figure 4:**
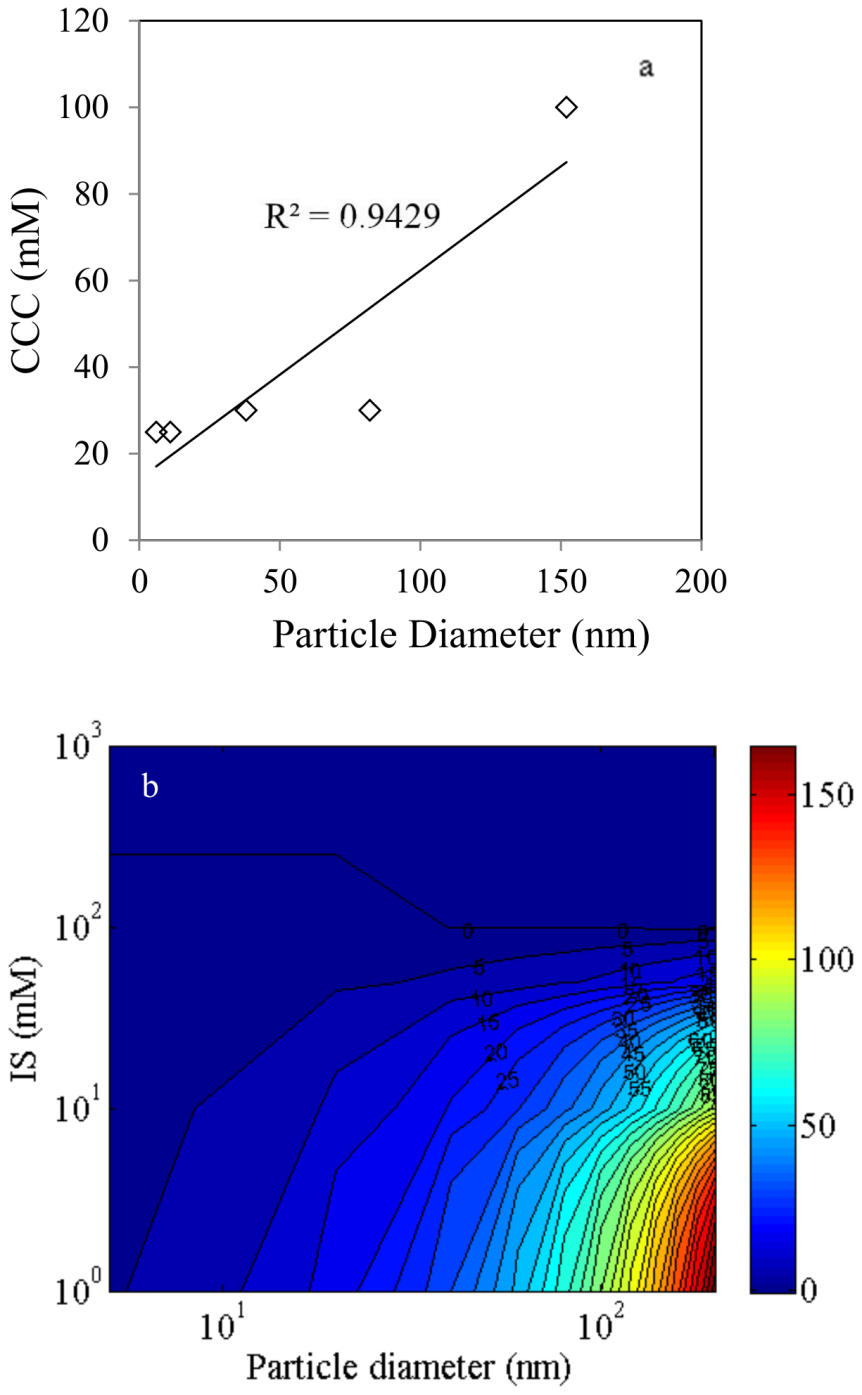
Anatase sphere CCC-particle size correlation and the theoretical prediction of the energy barrier. (a). Correlation between nanoparticle diameter and CCC for anatase sphere (AS) TiO_2_; (b). Predicted energy barrier contour map of TiO_2_ anatase nanospheres. The color bar denotes the energy barrier (unit kT).

Under the same solution chemistry, particle size has a clear effect on the stability of the TiO_2_ AS samples (Table 1). Smaller particles are much more prone to agglomerate; an ionic strength that is typical for surface water or groundwater [3] can completely destabilize the suspension. Larger AS particles exhibit larger CCC values. A linear correlation was found between particle size and CCC, with a R^2^ of 0.9429 (Figure 4a). A similar trend was reported for 12, 32, and 65 nm hematite nanoparticles [25]. According to DLVO theory, both van der Waals attraction and the electrostatic repulsion are functions of particle diameter (see Equation S1 and S2 in File S1). Therefore, a higher energy barrier is predicted as particle diameter increases. Figure 4b shows the contour map of energy barrier as a function of both particle size and the ionic strength. Assuming that a suspension is completely destabilized when the energy barrier is comparable to the thermo-kinetic energy (1 kT), indeed the CCC is predicted to increase as particle size increases (Figure 4b).

**Figure 5:**
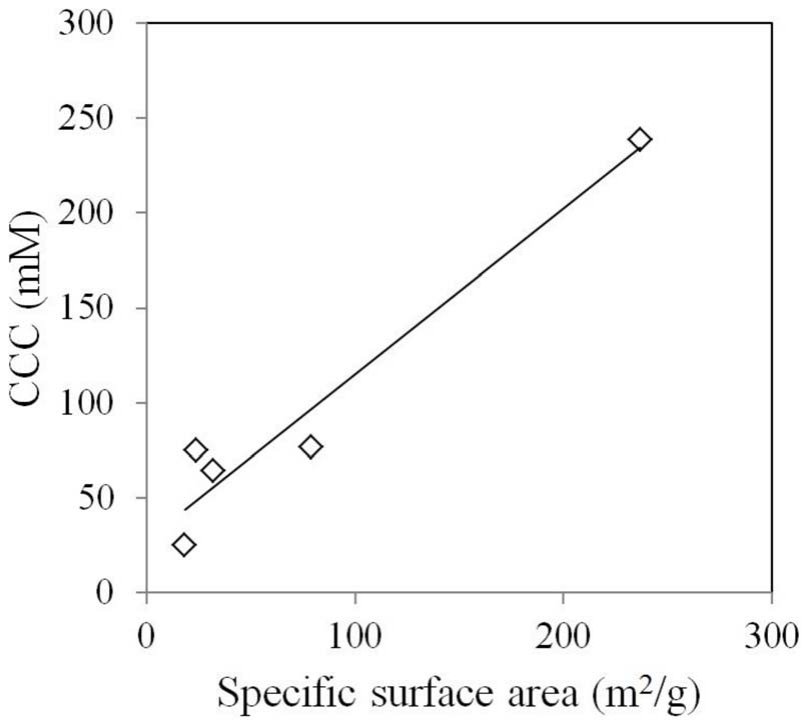
Correlation between specific surface area and CCC for rutile rods.

**Figure 6:**
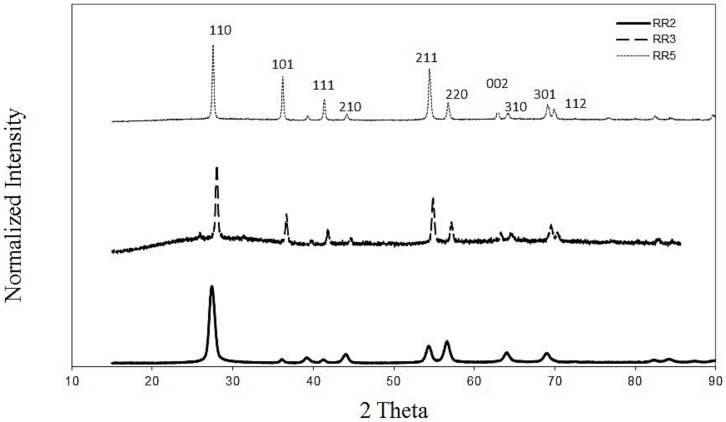
XRD spectra of RR2, RR3, RR4 (rutile rod) samples.

The same relationship does not hold for the rutile rods samples (Table 1, Figure S3 in File S1). The 15×4 nm rutile rods are the most stable among the five rods, with a CCC c.a. 200 mM NaCl. In contrast, only 25 mM NaCl is needed to induce DLCA aggregation for RR5, the largest rutile sample. A plot of the specific surface area against CCC revealed that a proportionality exists between the two parameters for rutile rods (Figure 5). A specific surface area - CCC correlation was reported previously for CdSe nanospheres and CdSe nanorods [26]. Qualitatively the DLVO theory predicts a linear proportionality between particle size and the energy barrier, provided all the other parameters identical (Equation S1 and S2). The observed opposite trend for the rutile rods samples suggests that bulk or surface properties other than the particle size may play a central role in the stability of rutile rods. As suggested by Onsager decades ago and reiterated by McBride and Bayeye recently, for clay suspensions, particle geometry has a strong influence on the colloidal properties due to the Covolume Effect [35]. The XRD spectra revealed a systematic increase in peak intensity along the crystal face [101], [111], [211] directions as the dimension of rutile rods increased (Figure 6, remaining XRD data presented in Figure S4 in File S1. This indicates a shift in exposed crystal face composition as rutile rod size changes. Various rutile crystal faces are known to possess different surface energies [36], [37], therefore changes in exposed crystal face composition may lead to altered surface energy and in turn influence colloidal stability. In addition, Abbas and her coworkers analyzed the effect of particle size on surface charge density for metal oxide nanoparticles using the corrected Debye-Huckel theory and Monte Carlo simulation [34]. Their results revealed that a considerable increase in surface charge density occurs when the particle diameter decreases. Such an increase in surface charge density may attribute to increased stability for smaller rutile rods.

### 3.4. Influence of humic acid

The colloidal stability of the TiO_2_ samples were tested in the presence of 10 mg/L Suwannee River natural organic matter (NOM), and the CCC values are reported in Table 1. In our previous study, 10 mg/L organic matter (humic acid) was found to significantly enhance the stability of the P25 TiO_2_ NPs [18]. In contrast, for most TiO_2_ samples in this study (except RR1), the effect of NOM in altering the CCC was minor (Table 1). Chen et al. showed that the adsorption of humic acid to TiO_2_ nanoparticles is pH dependent. At pH 5.7, humic acid significantly enhances the transport of TiO_2_; while at pH 9.0, only a small amount of humic acid adsorbs to the TiO_2_ surface [38]. Therefore, at the pH condition tested in this study (pH = 8), humic acid adsorption is likely to be small. Moreover, given the relatively large surface potentials of most of the TiO_2_ samples at pH 8 (Figure 2), even the adsorbed humic acid probably only led to minimal increase of surface charge and therefore minimal change in the CCC. Thus the potential for steric interference from adsorbed NOM was not very significant for most of these particles.

For RR1, the addition of 10 mg/L NOM actually shifted the CCC towards a much lower value. Given the large specific surface area of RR1 (236.4 m^2^/g, at least a factor of 3 larger than the rest of the samples), NOM probably could only partially cover the RR1 particles, or coat some particles and leave the remaining uncovered. In either case, there are available surfaces for a single humic acid macromolecule to attach to multiple nanoparticles; therefore the “bridging effect” may take place and facilitate aggregation [10], [20], [29]. The “bridging effect” of divalent cations is well-studied and –documented [39], [40]. In contrast, due to the low valence, the binding between monovalent cations and macromolecules are believed to be too weak to facilitate bridging. However, recent studies have shown both theoretically and experimentally that monovalent cations can indeed accelerate aggregation by bridging multiple nanoparticles [41], [42].

## Conclusion

We report here the distinctly different aggregation behaviors of a set of TiO_2_ nanoparticles with varying size, crystal structure, and morphology. The isoelectric points of both anatase spheres and rutile rods shift towards a lower pH value as the particle size increases. The *CCCs* of anatase spheres correlate well with particle size, which agrees with the DLVO prediction. In contrast, *CCCs* of rutile rods exhibit a strong dependence on the specific surface area, indicating it is the surface chemistry rather than the bulk properties that dominates rutile rods aggregation. Under the conditions tested, the effect of NOM in stabilizing most TiO_2_ samples was minor, since all the TiO_2_ samples already possessed a large negative charge without the presence of NOM.

Nanomaterials are increasingly engineered with varying properties in the pursuit of promising biological, medical, electronic, or environmental applications. Yet the effect of nanoparticles material property alteration in controlling their environmental fate and transport is largely under-investigated. This study has shown that nanomaterials with the same chemical composition, the same solution chemistry, but different size, shape, and crystal structure have different stability and mobility. Our results stress the need to accurately characterize the material properties, such as particle size, crystal structure, and specific surface area, for a reliable prediction of the aggregation behavior of nanoparticles.

## Supporting Information

File S1 **DLVO calculation.** Includes (Equations S1-S4) and Figures S1-S4. **Figure S1**. TEM/SEM images of AS4, AS5, RR4, and RR5 samples. Scale bar for RR4 is 100 nm. **Figure S2**. Representative agglomeration kinetics data for AS3, 50 mM NaCl. **Figure S3**. Critical Coagulation Concentration as a function of rutile rod length. **Figure S4.** XRD data for TiO2 samples (except RR2, RR3, RR5 are presented in Figure 6).
(DOCX)
