# Supplementary material for: Influence of Material Properties on TiO2 Nanoparticle Agglomeration
Source: PLoS One. 2013 Nov 25;8(11):e81239. doi: 10.1371/journal.pone.0081239 (PMC3839896; doi:10.1371/journal.pone.0081239)
Supplement: File S1 — DLVO calculation. Includes (Equations S1-S4) and Figures S1-S4. Figure S1. TEM/SEM images of AS4, AS5, RR4, and RR5 samples. Scale bar for RR4 is 100 nm. Figure S2. Representative agglomeration kinetics data for AS3, 50 mM NaCl. Figure S3. Critical Coagulation Concentration as a function of rutile rod length. Figure S4. XRD data for TiO2 samples (except RR2, RR3, RR5 are presented in Figure 6). (DOCX) [file pone.0081239.s001.docx]

**Supporting Information**

DLVO calculation

The van der Waals interaction is calculated by (Elimelech et al., 1995),

$V_{A}=-\frac{Aa_{1}a_{2}}{6h(a_{1}+a_{2})}$ Equation S1

Here *a_1_* and *a_2_* are particle radius, *h* is the separation distance, and *A* is the Hamaker constant. The electrostatic interaction between two spheres is calculated by,

$V_{R}=64\pi\frac{a_{1}a_{2}}{a_{1}+a_{2}}{(\frac{kT}{ze})}^{2}\gamma_{1}\gamma_{2}exp(-\kappa h)$ Equation S2

Here z is the indifferent ion valence, e is the elementary charge, κ is the Debye length, and γ is a dimensionless function of the surface potential, defined as,

$\gamma=tanh(\frac{ze\psi}{4kT})$ Equation S3

Where ψ is the surface charge, approximated by the zeta-potential of the particles. The total interaction energy is given by,

$V=V_{A}+V_{R}$ Equation S4


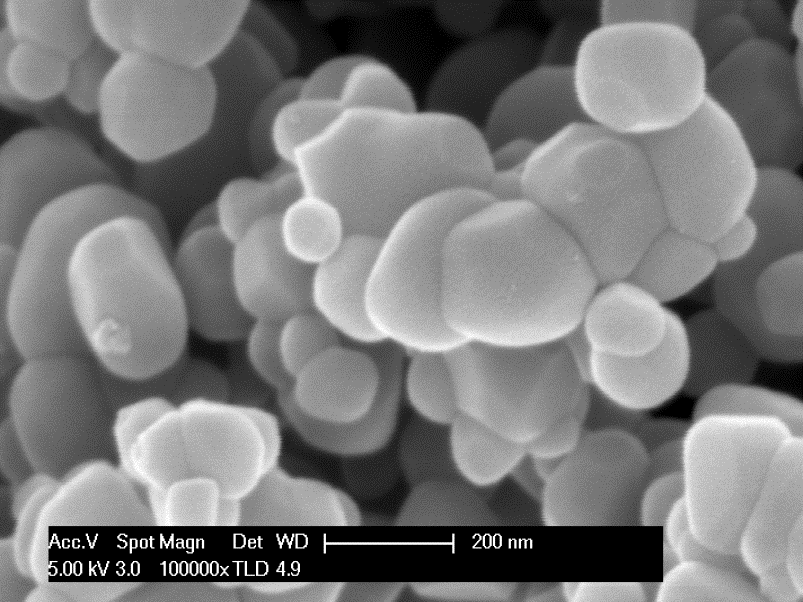


AS5


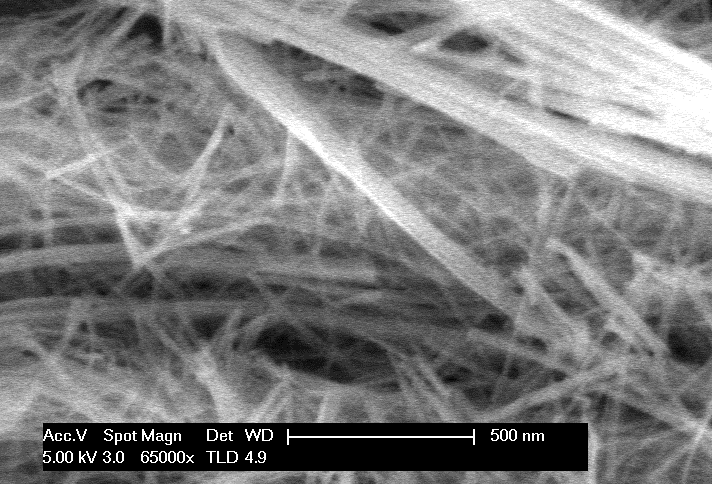

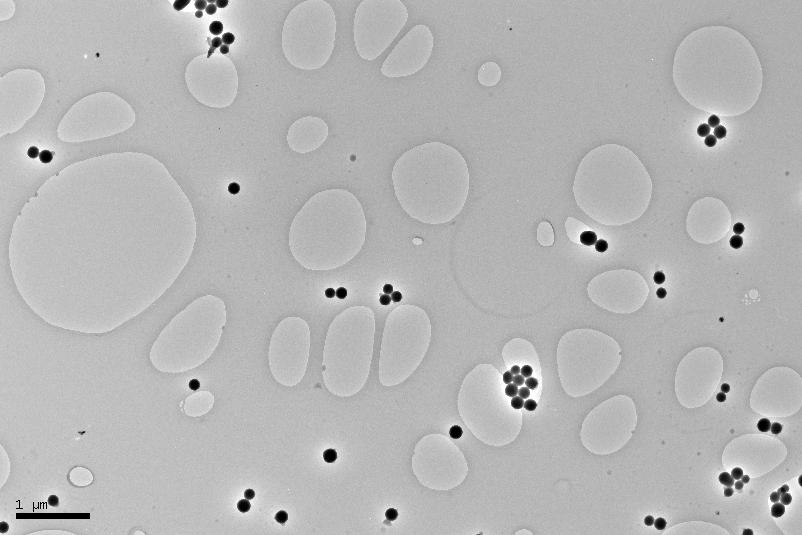

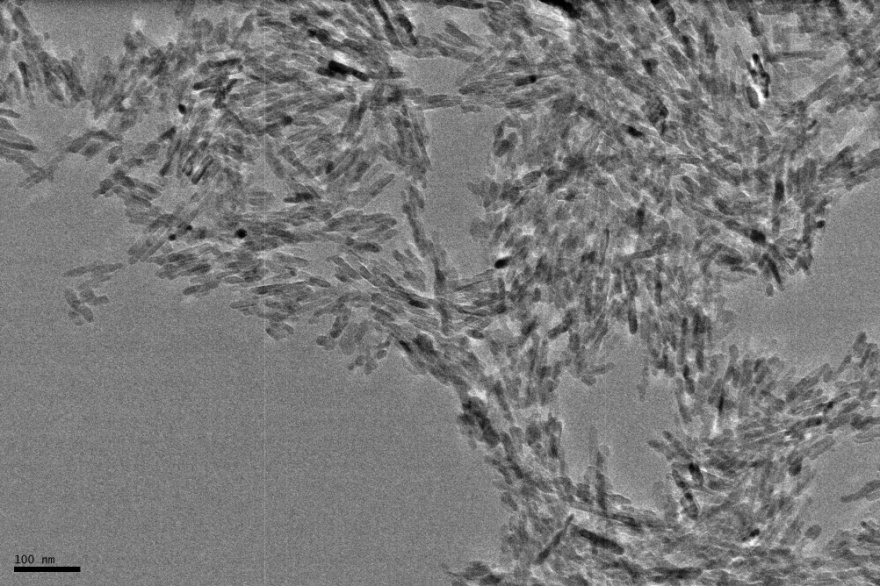


AS4

RR4

RR5

Figure S1. TEM/SEM images of AS4, AS5, RR4, and RR5 samples. Scale bar for RR4 is 100 nm.

Figure S2. Representative agglomeration kinetics data for AS3, 50 mM NaCl.


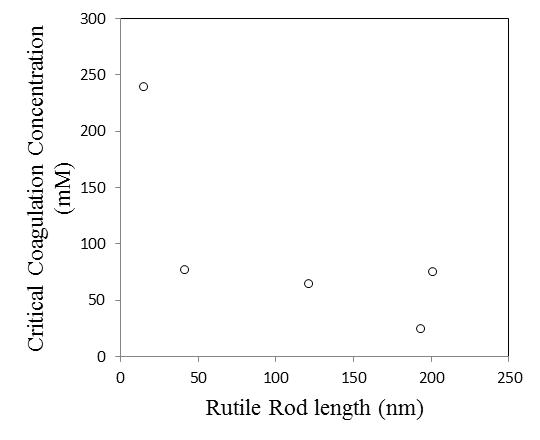


Figure S3. Critical Coagulation Concentration as a function of rutile rod length.


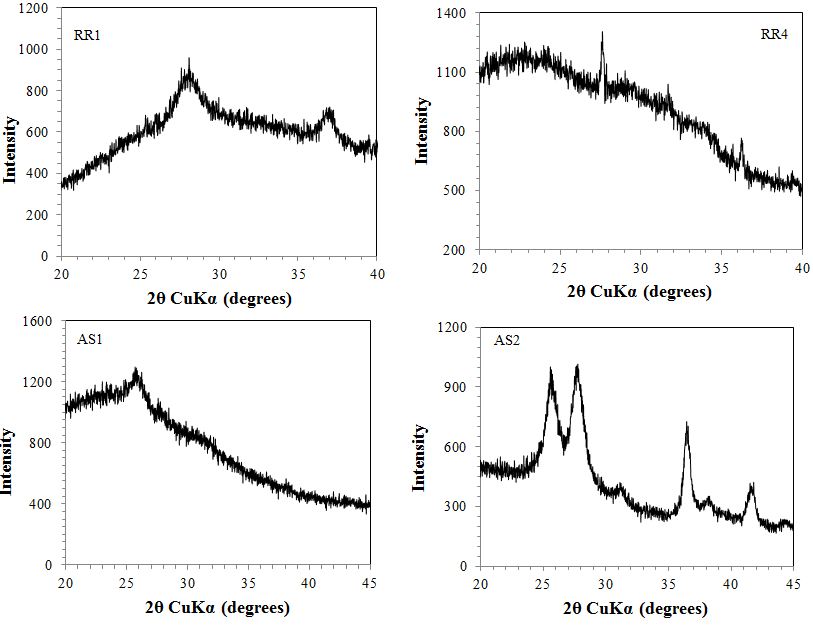

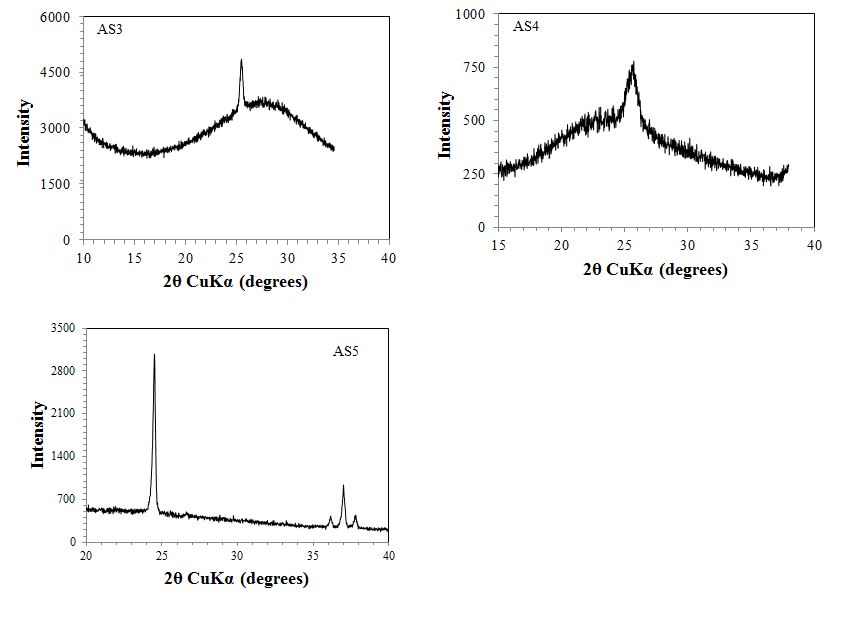


Figure S4. XRD data for TiO2 samples (except RR2, RR3, RR5 are presented in Figure 6).
